# Our New York Letter

**Published:** 1891-11

**Authors:** Wm. L. Russell

**Affiliations:** 151 East 50th St.; New York


### OUR NEW YORK LETTER.

---

NEW YORK, October 15, 1891.

At the last meeting of the Obstetrical Society an interesting discussion took place on the use of the forceps. A paper was read by Dr. Malcolm McLean, in which he stated that most cases which came to gynecologists traced their illness to child-bearing, and that operations "to assist nature," especially the use of the forceps, were largely responsible. He had seen many mistakes made in the course of an extensive experience, especially in regard to the time chosen for the application of the forceps, the manner of their application, and the kind of cases selected. Three cases were cited to illustrate these points.

The first was a primipara, aged twenty-six, who was confined at term. The presentation was normal, but the first stage was tedious. Eight hours after expulsive pains began the occiput was low down and in the second position. The patient then insisted on having assistance. Chloroform was given, she was put in the lithotomy position, the external genitals were washed with warm carbolized water, and the bladder emptied by means of a glass sterilized catheter. The forceps were then applied, and the occiput brought down well under the pubis. Two fingers of the left hand were then passed well back on the perineum, one on each side of the coccyx, and the head held in position by them while the forceps were removed. The pains then lifted the head out of the pelvis gradually. This was his usual method of procedure in all such cases. The child was breathing feebly and the heart was weak, and in spite of every effort it died. The birth was normal in every respect except for the delay, but the mother became an invalid from pelvic peritonitis. In this case the forceps should have been used hours before they were. Such instances were not common in these days, the opposite procedure being much more frequent.

The second case was also a primipara, aged twenty-two, who had had an uneventful pregnancy. Labor began at 10 A. M., and at 3 P. M. the os was dilated. At 4 P. M. the long Elliot forceps were applied by the attending physician with great difficulty, and efforts made to deliver for three hours. Dr. McLean was then called, and found the head firmly impacted in the swollen tissues at the superior strait. The Farrier-Lusk forceps were applied, and delivery effected with great difficulty. In this case the forceps were manifestly applied too early.

The third case illustrated the use of the forceps in a case to which they were not properly applicable. The woman was a primipara, aged thirty, who was in labor at term. The first stage was tedious, and after the second had lasted several hours there had still been no descent. The attending physician then applied the forceps, the blades passing with difficulty, and locking being hard to effect. Traction was then made, but the head did not descend. Other forceps were tried, the first having slipped off but without avail. After two hours had been spent in useless effort, Dr. McLean was called. He found a well-formed roomy pelvis, into which he introduced his hand and by feeling the child's ears discovered that he had an occiput posterior presentation to deal with. Rotation was easily effected, when the head settled down in the pelvis, and delivery was completed by the forceps. In this case if the hand had been introduced and the ears felt before forceps were applied much trouble would have been avoided. The forceps should never be applied at the superior strait without the introduction of the hand to ascertain the position of the head positively.

He did not regard restlessness in the first stage as a reason for the use of the forceps—nature was to be assisted, not violated. It was important to know just where to use the instruments and also how to use them. When the head was well down the blades could be removed, the fingers on each side of the coccyx grasping the head and holding it in position, and even lifting it from the pelvis. Rapid delivery was seldom necessary, and it was much better to be deliberate. The forceps were sometimes applied where version would be better because of disproportions. Nine times out of ten, however, version was not attempted until the forceps had failed, and thus the operation was rendered more difficult and was often complicated by lacerations of the uterus and vagina.

Dr. R. A. Murray agreed with Dr. McLean regarding the advisability of introducing the hand to ascertain the exact position of the head before applying the forceps. When the head was found movable above the brim the forceps were seldom applicable. When the cervix was not well receded over the head it was necessary to be careful to apply the forceps during the interval between the pains, to avoid injury to cervix.

Dr. Hanks believed that the forceps should never be applied when the head was movable above the brim. In such a case there was no indication for interference unless there was fever. In cases of occiput posterior position he had been able to make a diagnosis from the sutures and fontanelles, and had been able to accomplish rotation.

Dr. Chas. Jewett, of Brooklyn, stated that bad tears of the perineum were seldom observed in natural labors, but were usually the result of the forceps deliveries. In occiput posterior cases the position should be corrected early. He had succeeded in doing this in some cases, only by carrying the hand past the head to the shoulder. This was quite possible with antiseptic methods. He thought that rapid delivery with the forceps was too common, and was very bad. The indications for the forceps were, in general terms, any danger to the life or health of the mother or child.

Dr. Buckmaster thought that a good indication for the use of the forceps was the failure of the head to recede between the pains.

In closing the discussion Dr. McLean expressed his agreement with Dr. Buckmaster in regard to the non-recession of the head. It was bad to allow the head to remain fixed in one position for any great length of time. Another point in regard to the use of the forceps was in the amount of force used. This was often greater than was necessary, if care be taken to allow the head to choose its own position, by holding the wrist loose, so as to allow the instruments to turn with the head.

THE TREATMENT OF EPILEPSY.

This bugbear of medical science was again under discussion at the last meeting of the Academy. Dr. B. Sachs introduced the surgical side of the question. He stated that much had been expected from surgical treatment, but little had been realized. Statistics on the subject were valueless, the failures not having been recorded and many of the apparent successes having been announced too early.

In considering the question of operation in any case, it must be remembered that epilepsy was in many, if not most, cases not idiopathic. He examined very carefully for signs of infantile paralysis which had recovered and for a history of some injury to the brain or skull. Since doing so he recorded fewer idiopathic cases. Recent pathological investigations also showed that, though the initial canal lesion could not always be discovered it was quite common to find a resultant sclerosis. This was especially true of cases of localized spasm, Jacksonian epilepsy. The initial lesion was usually a meningeal hemorrhage or an area of softening caused by an embolism, these resulting eventually in a localized sclerosis. These conditions were in most instances found in the cortex. Where such lesions could be ascertained to exist, an operation for removal of the diseased area might be performed if the epilepsy were of the Jacksonian type, so as to render possible the location of the lesion. In children the function of the removed centre might be assumed by other parts of the brain ; in adults, however, paralysis was inevitable, and the choice would have to be made by the patient. Cure of epilepsy could not be predicted in any case.

The prevention of traumatic epilepsy was a promising field for the operator. An immediate exploratory operation should always be made in cases of injury to the skull. If depressed bone were discovered its removal should be accomplished by the trephine. These operations on the cranium could hardly be regarded as dangerous under present surgical methods. Seven cases of epilepsy had been operated on by his advice. In all the operation was quite extensive, and there was not a single death. These were in adults ; the result was different in children, three out of four cases which he had had operated upon having died.

Dr. C. L. Dana then took up the medical treatment. He said that, in view of the recent exhaustive review of the subject by Dr. Seguin, he would not go over the whole field but would confine himself mainly to some points in diagnosis which were useful as guides to treatment. Many epileptics showed signs of a chronic degenerative condition which rendered treatment hopeless. It was apparent in some cases in the general expression, while in others at the other end of the scale the epilepsy seemed but an accident. Certain stigmata, or marks, of the degenerative type should always be looked for. These were, in brief, asymmetry of the cranium, shortening of the parietal or the frontal arches, a sharp palatal arch, and badly set teeth, difference in the color of the eyes and in the position of the pupils, deformities of the ears from absence of the helix or from being badly placed, abnormally long fingers and a marked difference in the size of the two hands or feet, deviation of the nose, and flattening of the nasal bones. Sexual anomalies were common, and irregular innervation in voluntary movements. Irresistible erotic tendencies and masturbation, irritability, viciousness and a lack of self-control were physical conditions sometimes noticed. Just in proportions as these features predominated was the hopelessness of treatment.

Hydrotherapy and mental occupation were of more real value than the bromides. The former was not sufficiently understood in this country. The use of phosphoric acid was a useful adjunct, as careful observation had shown that the excretion of phosphates was diminished in these patients. The use of antipyrine had been lately recommended instead of chloral as advised by Dr. Seguin.

Dr. J. A. Wyeth continued the discussion by describing the different methods of exposing the brain. He also stated that the operation could not be regarded as serious if performed under careful antisepsis.

Dr. W. R. Birdsall believed that the danger was much greater when portions of the cortex were removed. It was too soon, however, to decide on the value of these operations, as improved surgical methods were of so recent date. We were still in the experimental stage. He relied on the bromides with adjuvants such as the mineral acids and other tonic remedies. Numerous modes of treatment had been employed in the treatment of this disease. In 1800 a folio book of a hundred and fifty pages was given up entirely to the enumeration of those tried up to that date. The list had been lengthened since. As late as 1850, the menstrual blood of a virgin was seriously recommended. Operative procedures of various kinds had been tried, such as amputation of an arm, castration, and ligation of a carotid. The influence of mental impressions was almost as great on epileptic as on hysteria cases, and they improved after any operation or even change of treatment.

Dr. M. Allen Starr thought that operations should be confined to traumatic cases, and those in which the location of the brain lesion could be definitely located. Unfortunately, the majority of epileptics did not belong to these classes. He never advised excision of a portion of the cortex. Unless the area of sclerosis were such as could be distinctly defined by means of an ordinary magnifying glass, the scar left by the removal of a portion of the brain would result in a more serious irritation.

He relied on the bromides. Recent investigations had shown that increase of uric acid in the urine often preceded a convulsive attack which could be prevented by appropriate treatment by alkalies, etc. It order to take advantage of this indication daily examination of the urine was necessary. He had been able to have this done in one case by a physician who was a victim of the disease, and it seemed as though there was truth in the statement and value in the treatment.

THE ABUSE OF MEDICAL CHARITY.

Some interesting facts pertaining to this subject were set forth in a paper read by Dr. W. H. Bates, at the last meeting of the Society of Medical Jurisprudence. It was stated that during the year 1890, the hospitals and dispensaries of New York treated 550,000 new cases, not to speak of the thousands treated by the Health Department. The same large proportion was noticeable in the other large cities. It was said that one-half the population of London asked for and received gratuitous medical treatment, and as large a proportion of the inhabitants of Edinburgh. The ratio was constantly increasing. Many persons sought dispensary treatment who were able to pay a physician a reasonable fee. Actual investigation had shown that forty per cent. of dispensary patients lie regarding their address, or their financial condition or both. This was a great wrong to the deserving poor, whose circumstances required that they seek aid at the dispensaries. The treatment received was certainly inefficient where from thirty to sixty patients were disposed of in an hour by one man, which was not unusual in the large dispensaries here.

The remedy suggested was the passage of a law making it a misdemeanor for a person to seek to obtain gratuitous medical treatment through misrepresentation. During the discussion of this suggestion the astounding statement was made and seemed to be generally agreed to, that the passage of such a bill would be opposed by the trustees of the dispensaries and hospitals, who occupied their position for some selfish end, and would not agree to anything that would diminish the number of cases treated.

WM. L. RUSSELL.

151 *East 50th St.*

